# Epstein–Barr virus and malaria upregulate AID and APOBEC3 enzymes, but only AID seems to play a major mutagenic role in Burkitt lymphoma

**DOI:** 10.1002/eji.202249820

**Published:** 2022-05-12

**Authors:** Andrea M. Summerauer, Vera Jäggi, Rodney Ogwang, Sabrina Traxel, Lorenzo Colombo, Eivind Amundsen, Tatjana Eyer, Bibin Subramanian, Jan Fehr, Pierre-Yves Mantel, Richard Idro, Simone Bürgler

**Affiliations:** 1 Experimental Infectious Diseases and Cancer Research, Children’s Research Center, University Children’s Hospital Zurich, Zurich, Switzerland; 2 Department of Infectious Diseases and Hospital Epidemiology, University Hospital Zurich, Zurich, Switzerland; 3 College of Health Sciences, Makerere University, Kampala, Uganda; 4 Centre of Tropical Neuroscience, Kitgum Site, Kampala, Uganda; 5 KEMRI-Wellcome Trust Research Programme, Centre for Geographic Medicine Coast, Kilifi, Kenya; 6 KG Jebsen Centre for B Cell Malignancies, Institute of Clinical Medicine, University of Oslo, Oslo, Norway; 7 Department of Oncology, Microbiology, and Immunology, Faculty of Science and Medicine, University of Fribourg, Fribourg, Switzerland

**Keywords:** APOBEC, Burkitt lymphoma, Epstein–Barr virus, malaria, mutagenesis

## Abstract

Endemic Burkitt lymphoma (eBL) is characterized by an oncogenic IGH/c-MYC translocation and Epstein–Barr virus (EBV) positivity, and is epidemiologically linked to Plasmodium falciparum malaria. Both EBV and malaria are thought to contribute to eBL by inducing the expression of activation-induced cytidine deaminase (AID), an enzyme involved in the IGH/c-MYC translocation. AID/apolipoprotein B mRNA editing catalytic polypeptide-like (AID/APOBEC) family enzymes have recently emerged as potent mutagenic sources in a variety of cancers, but apart from AID, their involvement in eBL and their regulation by EBV and P. falciparum is unknown. Here, we show that upon inoculation with EBV, human B cells strongly upregulate the expression of enzymatically active APOBEC3B and APOBEC3G. In addition, we found significantly increased levels of APOBEC3A in B cells of malaria patients, which correlated with parasite load. Interestingly, despite the fact that APOBEC3A, APOBEC3B, and APOBEC3G caused c-MYC mutations when overexpressed in HEK293T cells, a mutational enrichment in eBL tumors was only detected in AID motifs. This suggests that even though the EBV- and P. falciparum-directed immune response triggers the expression and activity of several AID/APOBEC members, only the upregulation of AID has oncogenic consequences, while the induction of the APOBEC3 subfamily may primarily have immunoprotective functions.

## Introduction

Endemic Burkitt lymphoma (eBL) is driven by the translocation of the *c-MYC* gene under the control of immunoglobulin (*IG*) gene enhancers, leading to elevated c-Myc activity and, in conjunction with other driver mutations, to increased cell proliferation and reduced apoptosis [1, 2]. Even though it is well established that virtually all eBL cells carry the Epstein–Barr virus (EBV), its exact role in eBL pathogenesis remains unclear [3]. Similarly, despite the long-known epidemiologic link between eBL and *Plasmodium falciparum* malaria, the underlying mechanism remains poorly understood [4].

Activation-induced cytidine deaminase/apolipoprotein B mRNA editing catalytic polypeptide-like (AID/APOBEC) enzymes deaminate cytidines in RNA and/or DNA. The resulting uracils are subsequently processed by repair proteins in an either high- or low-fidelity manner, with the latter resulting in mutations [5, 6]. In DNA stretches with high uracil density, the repair machinery may further induce double-strand breaks, leading to (aberrant) DNA recombination or translocations. The human AID/APOBEC family comprises 11 members: AID, APOBEC1, APOBEC2, APOBEC3 with its subfamily members APOBEC3A, APOBEC3B, APOBEC3C, APOBEC3D/E, APOBEC3F, APOBEC3G, APOBEC3H, and APOBEC4. While AID plays a key role in somatic hypermutation and class-switch recombination by deaminating *IG* genes [7], APOBEC3 enzymes are important in restricting the replication of viruses by acting on viral DNA or RNA. In fact, each of the seven human APOBEC3 enzymes has been implicated in the restriction and mutation of a variety of different human viruses including retroviruses, parvoviruses, herpesviruses, papillomaviruses, hepatitis B virus, and retrotransposons [8, 9]. APOBEC3G, for example, seems to play a key role in restricting HIV-1 [8, 9], while APOBEC3C restricts HSV replication [10]. EBV, in turn, seems to be mainly affected by APOBEC3B [11, 12]. Interestingly, APOBEC3B may also be involved in the immune response against *P. falciparum*, since the absence of the *APOBEC3B* gene was found to be associated with susceptibility to *P. falciparum* malaria [13].

Beside these immunoprotective functions, AID/APOBEC enzymes are known to trigger oncogenic point mutations or translocations in host genes, thereby contributing to the development and progression of T- and B-cell malignancies [14–17] as well as solid tissue cancers [18–22]. Despite the undebatable role of APOBEC3 enzymes in host defense and the recent emergence of AID/APOBEC enzymes as potent sources of oncogenic mutations in a wide range of malignancies, information on the exact role of the individual members and the mechanisms regulating their expression and activity in different cell types is still sparse. AID, the most extensively studied family member, has been shown to catalyze the *IGH*/*c-MYC* translocation that drives eBL [23, 24]. Since both EBV [25, 26] and *P. falciparum* [27, 28] upregulate AID expression, they may contribute to the *IGH*/*c-MYC* translocation and mutations in other eBL-associated genes by causing an immune response that leads to enhanced (off-target) AID activity. The involvement of further AID/APOBEC enzymes in eBL pathogenesis and progression is unclear. In this study, we addressed the regulation of AID/APOBEC enzyme expression and activity by EBV and *P. falciparum* in vitro and ex vivo, and analyzed their potential mutagenic potential in eBL.

## Results

### EBV induces AICDA, APOBEC3B, and APOBEC3G mRNA expression in human B cells

**Figure 1:**
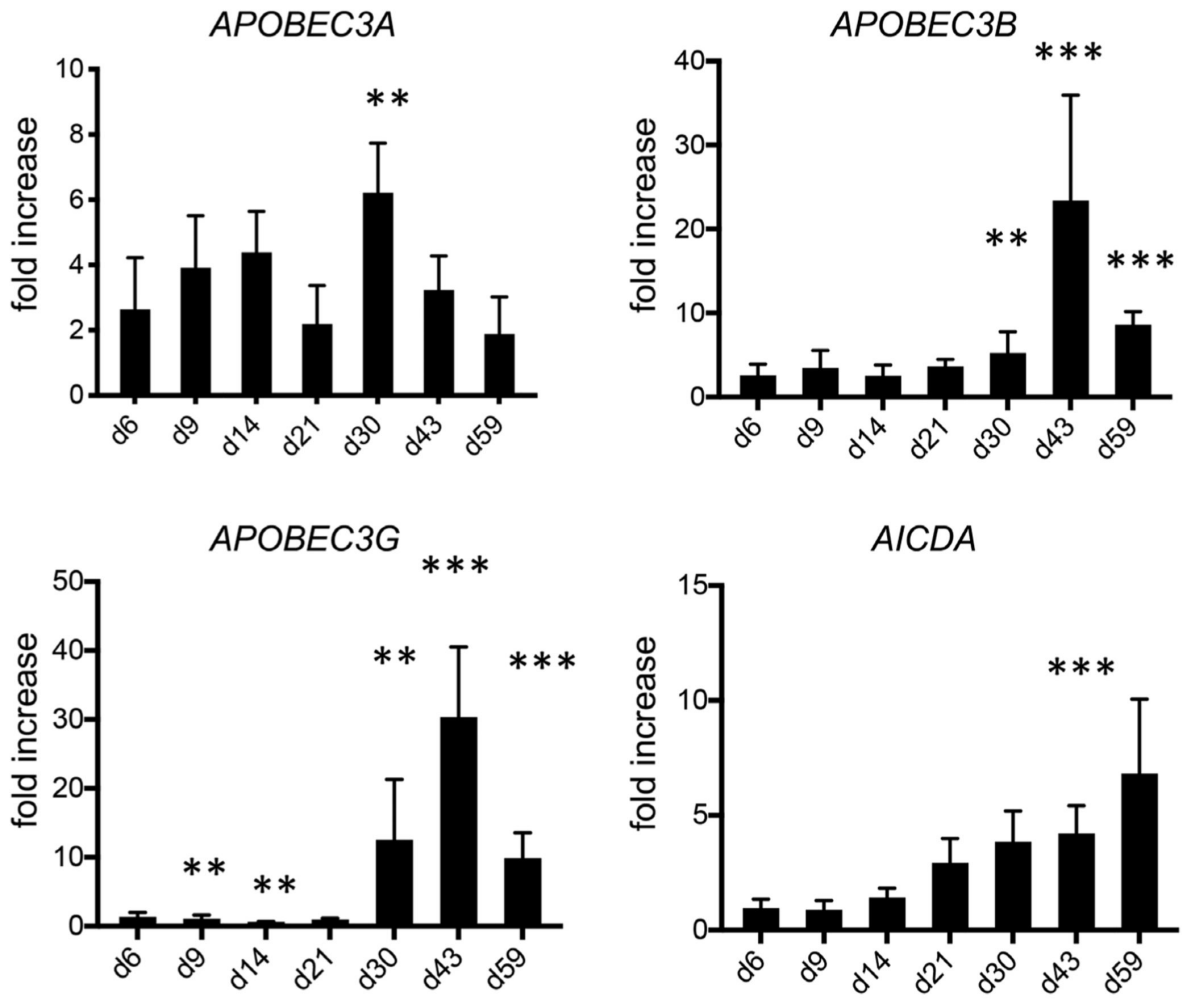
EBV induces mRNA expression of *AICDA*, *APOBEC3B*, and *APOBEC3G* in human B cells. Tonsillar B cells from 12 donors were inoculated with EBV and expression of AID/APOBEC enzymes was determined by qRT-PCR. Shown is the fold increase compared to expression in noninfected B cells of the same donor. Data are shown as mean ± SD (n = 12) from 12 independent experiments. *p* values were determined using Wilcoxon matched-pairs signed rank test with Bonferroni correction for multiple comparison. **p < 0.01, ***p < 0.001.

Knowing that EBV enhances AID expression in human B cells [7, 29], we wondered whether other AID/APOBEC enzymes are upregulated by EBV as well. To test this, we inoculated human tonsillar B cells with EBV and analyzed expression of AID/APOBEC enzymes by qRT-PCR at various time points. Besides confirming the upregulation of *AICDA*, we observed a strong enhancement of *APOBEC3B* and *APOBEC3G* expression, while *APOBEC3A* was only slightly upregulated (Fig. 1). No changes were observed for *APOBEC1*, *APOBEC3C*, *APOBEC3D/E*, *APOBEC3F*, and *APOBEC3H* levels (Supporting information Fig. S1). EBV-induced mRNA expression of *APOBEC3B* and *APOBEC3G* peaked between days 43 and 59, similar to what we and others [29, 30] have observed for *AICDA*.

### EBV induces the enzymatic activity of APOBEC3A/B and APOBEC3G in human B cells

**Figure 2:**
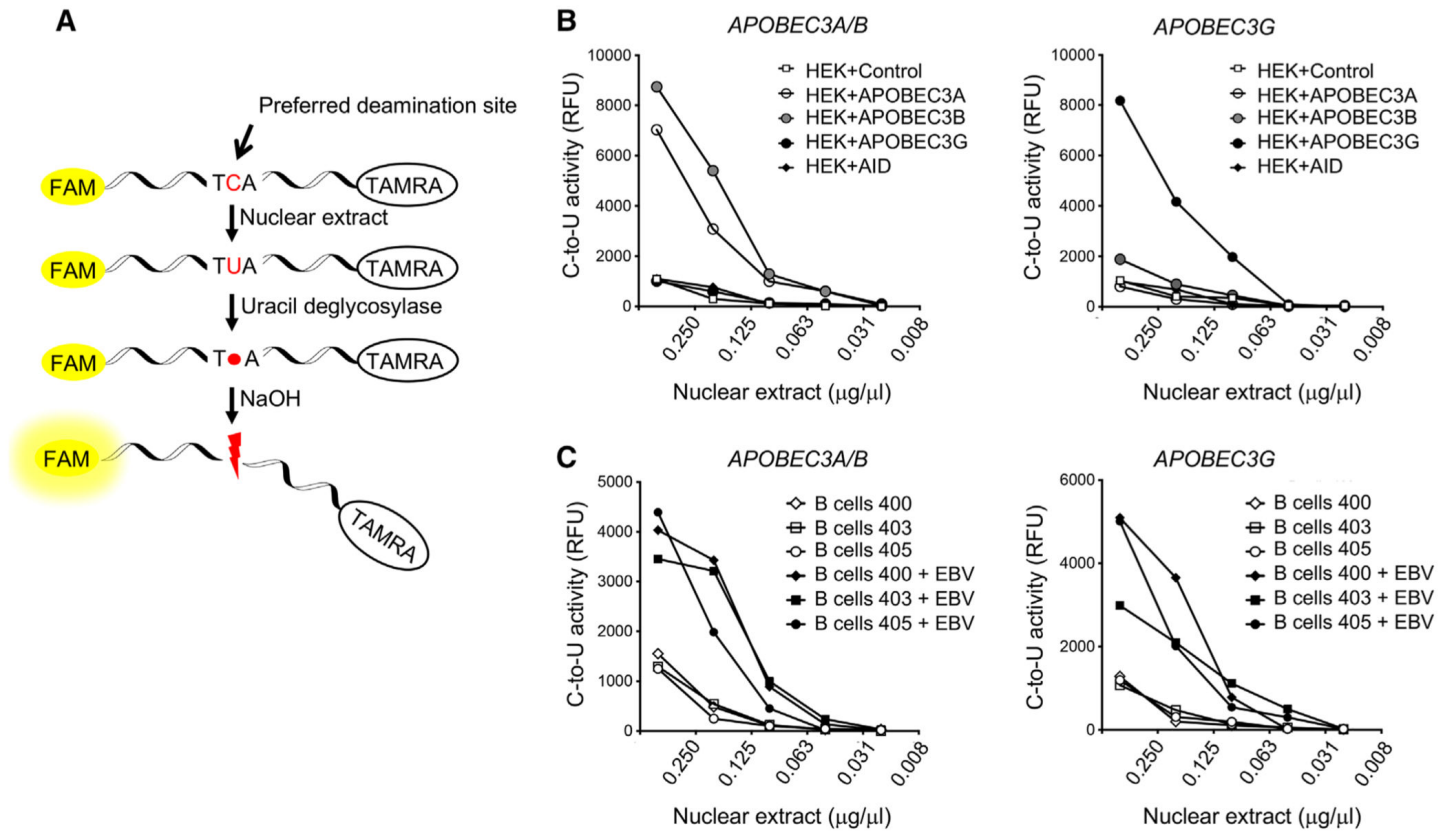
EBV induces APOBEC3A/B and APOBEC3G activity in human B cells. (**A)** Principle of the in vitro deaminase activity assay: An oligonucleotide containing the preferred deamination site for a given AID/APOBEC enzyme, a fluorescent dye (FAM), and a quencher (TAMRA) is incubated with nuclear extracts of the cells of interest. If the corresponding AID/APOBEC enzyme is expressed and active, the cytidine is deaminated. Subsequent addition of uracil deglycosylase generates an abasic site that is cleaved by addition of NaOH, leading to the release of the FAM dye, and enzymatic activity can be assessed as a function of fluorescence. (**B)** Establishment of the in vitro deaminase activity assay for APOBEC3A/B and APOBEC3G. HEK293T cells were transfected with APOBEC3A, APOBEC3B, APOBEC3G, AID, or a control vector, and deamination of cytidines within the consensus sequence for APOBEC3A/B (left panel) and APOBEC3G (right panel) was determined by deaminase activity assays. **(C)** Deaminase activity of APOBEC3A/B (left panel) and APOBEC3G (right panel) in B cells of three donors before and at day 43 after EBV inoculation was determined by deaminase activity assays. RFU, relative fluorescence units. (B and C) Data shown are from nine independent experiments.

Due to their high sequence homology, a specific quantitative detection of individual AID/APOBEC enzymes using antibodies is hardly feasible. Thus, we took advantage of the fact that most of the enzymes target a distinct DNA motif, and measured changes in enzyme-specific deaminase activity using an in vitro deaminase activity assay instead (Fig. 2). First, we validated the specificity of the assay by transfecting HEK293T cells with APOBEC3A, APOBEC3B, APOBEC3G, AID, or a control vector, respectively (Fig. 2A and B). Nuclear extracts of the transfected cells were then incubated with oligonucleotides containing the preferred deamination site for the enzymes of interest, and enzymatic activity was analyzed. Since the preferred deamination sequence for APOBEC3A and APOBEC3B is not fully distinct, we used a sequence recognized by both enzymes (termed APOBEC3A/B hereafter). Even though a deaminase activity assay has previously been established for mouse AID [31], the deamination by human AID in our assay was not totally sequence-specific. Importantly, however, the assay enabled us to determine DNA deamination by APOBEC3A and/or APOBEC3B and by APOBEC3G, while ruling out a contribution of AID. To assess whether EBV induces the expression of enzymatically active APOBEC3A and/or APOBEC3B and APOBEC3G in B cells, nuclear extracts of B cells from three donors were isolated before and 43 days after EBV inoculation, the purity of the cellular fractionation was confirmed by Western blot (Supporting information Fig. S2) and nuclear extracts were tested for APOBEC3A/B and APOBEC3G activity (Fig. 2C). In fact, we found a higher APOBEC3A/B and APOBEC3G activity in EBV-transformed B cells compared to autologous noninfected B cells from the same donor. This demonstrated that EBV induced nuclear expression of enzymatically active APOBEC3A and/or APOBEC3B and APOBEC3G.

### APOBEC3A expression is increased in B cells of malaria patients and correlates with parasite load

**Figure 3:**
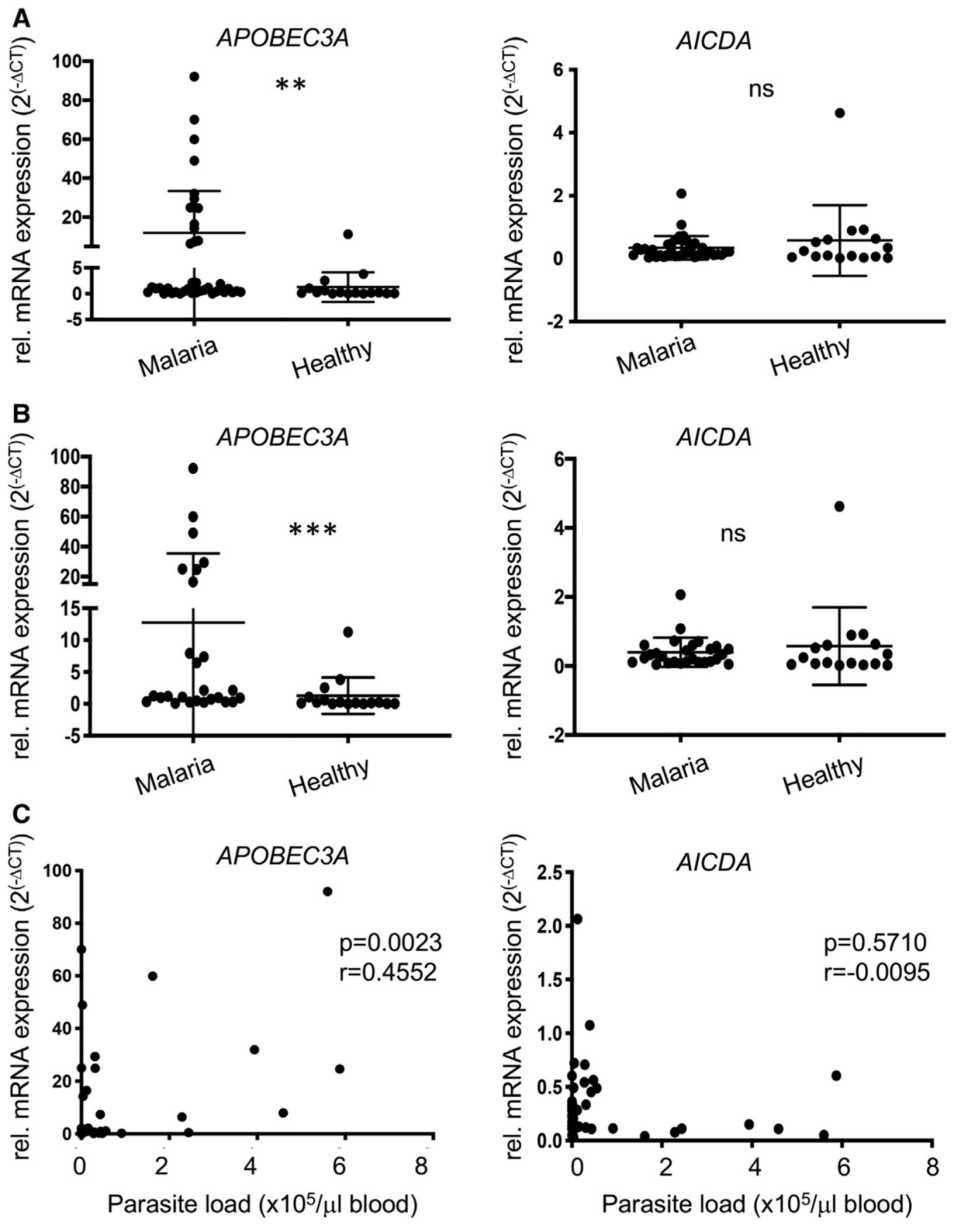
*Plasmodium falciparum* induces expression of APOBEC3A in B cells. (**A** and **B)** Expression of *APOBEC3A* and *AICDA* in B cells of malaria patients and age-matched healthy controls was measured by qRT-PCR. Dots represent individual donors, bars represent mean ± SD, ΔCT = CT^A3A or ACIDA^-CT^HBMS^. *p* values were determined using Mann-Whitney test. **p < 0.01, ***p < 0.001, ns = not significant. (**A)** Expression values of all evaluable patients (N = 40). **(B)** Expression values of patients without antimalarial treatment (N = 27). (**C)** Expression of *APOBEC3A* (top panel) and *AICDA* (lower panel) in B cells of malaria patients (N = 40) versus blood parasite concentration. Dots represent individual donors, bars represent mean ± SD, ΔCT = CT^A3AorACIDA^-CT^HBMS^. Data shown are from three independent experiments. Statistical parameters were determined using Spearman test.

Next, we turned our attention to *P. falciparum*, the second pathogen closely linked to eBL pathogenesis. To address whether *P. falciparum* infection may upregulate further AID/APOBEC enzymes, we analyzed mRNA expression of AID as well as APOBEC3 family enzymes in peripheral blood B cells of pediatric patients with acute malaria and compared the levels to the expression levels in B cells of age- and sex-matched healthy controls (Fig. 3A and (Supporting information Fig. S3). While we did not observe any difference in expression levels for *AICDA* and *APOBEC3B-H*, *APOBEC3A* levels were significantly increased in malaria patients (*p* = 0.0023). Importantly, when excluding patients who had been treated with antimalarial drugs, this upregulation was even more significant (*p* = 0.0004; Fig. 3B). In agreement with this finding, *APOBEC3A* levels strongly correlated with blood parasite concentration (*p* = 0.0023; Fig. 3C). This suggested that *P. falciparum* parasites may upregulate *APOBEC3A* expression in peripheral blood B cells.

### Human B cells upregulate APOBEC3A expression upon stimulation with extracellular vesicles of *P. falciparum*-infected erythrocytes

**Figure 4:**
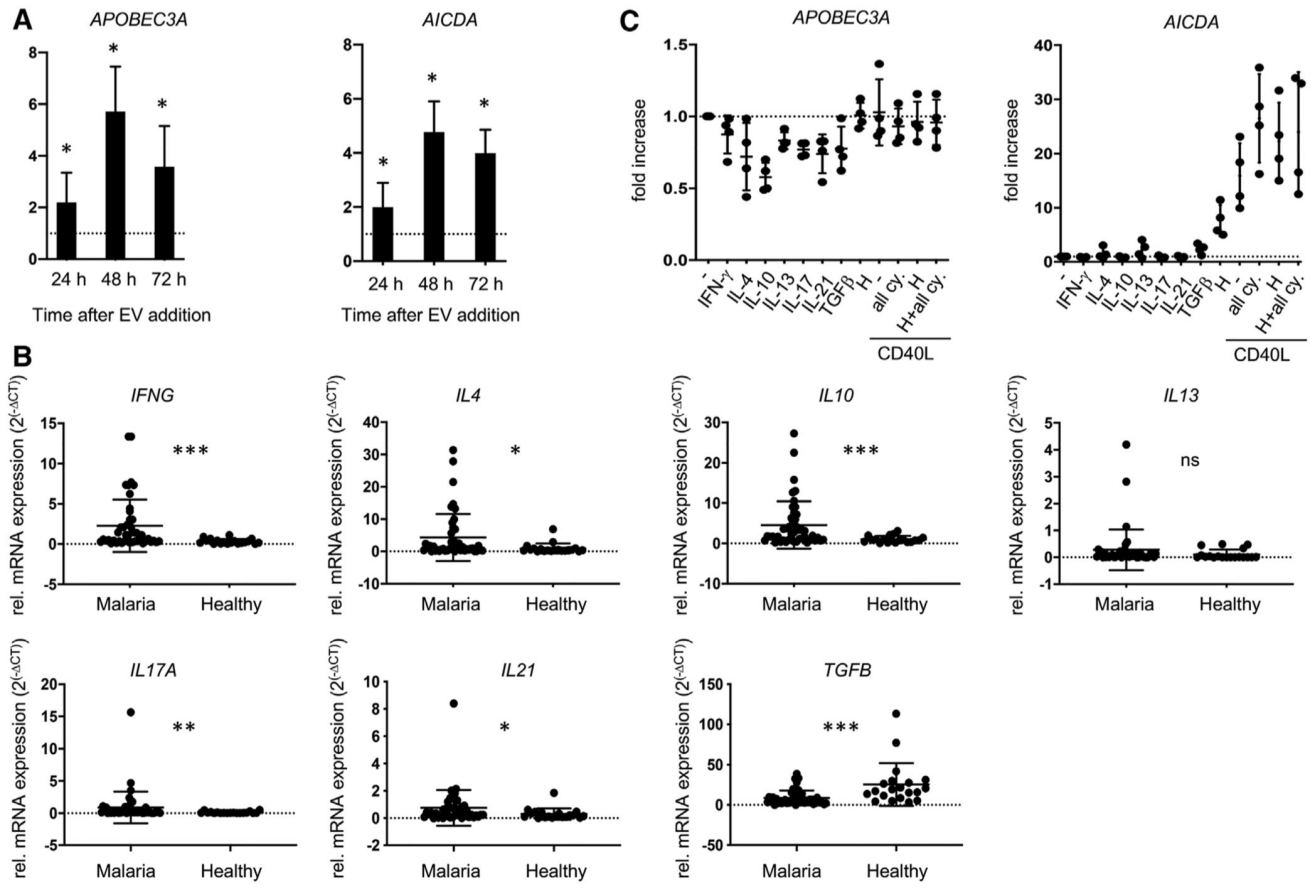
*Plasmodium falciparum* induces the expression of *APOBEC3A* in B cells. **(A)** B cells from peripheral blood of healthy donors were stimulated with 10 μg/mL extracellular vesicles of *P.falciparum*-infected (iEV) or noninfected (niEV) erythrocytes for the indicated time points, and expression of AID/APOBEC enzymes was determined by qRT-PCR. Shown is the fold increase in iEV-stimulated B cells compared to expression at day 0, normalized to niEV-stimulated B cells (mean ± SD of six donors). *p* values were determined using Wilcoxon matched-pairs signed rank test with Bonferroni correction for multiple comparison, *p < 0.05. (**B)** Cytokine expression in Th cells of malaria patients and age-matched healthy controls was measured by qRT-PCR. Dots represent individual donors, bars represent mean ± SD (N = 40), ΔCT = CT^cytokine^-CT^HBMS^.*p* values were determined using Mann-Whitney test. *p < 0.05, **p < 0.01, ***p < 0.001; ns, not significant. (**C)** Peripheral blood B cells were stimulated for 48 h, as indicated, and expression of *APOBEC3A* and *AICDA* was determined by qRT-PCR. Shown is fold increase to expression in nonstimulated cells (mean ± SD of four donors); all cy = IFN-y + IL-4 + IL-10 + IL-13 + IL-17 + IL-21 + TGF-p; H, hemozoin. Data shown are from three independent experiments.

To address the mechanism of malaria-induced *APOBEC3A* expression in B cells, we stimulated B cells from healthy donors with extracellular vesicles (EV) of *P. falciparum*-infected erythrocytes. Such vesicles display the same molecules on their surface as *P. falciparum*-infected erythrocytes and are known to activate immune cells in human and rodent malaria, thus, mimicking the interaction of B cells with infected erythrocytes in malaria patients [32–35]. Vesicles from noninfected erythrocytes served as a control. In fact, B cells stimulated with vesicles of *P. falciparum*-infected erythrocytes expressed higher levels of *APOBEC3A* and *AICDA* compared to B cells stimulated with control vesicles, while no differences in expression levels were observed for *APOBEC3B-H* (Fig. 4A). Nevertheless, the upregulation was not very profound, and we wondered whether *APOBECA* upregulation in malaria patients’ B cells might be caused by an indirect mechanism, for example, via Th cell-derived stimuli such as cytokines. To obtain candidate cytokine involved in malaria-induced APOBEC3A upregulation, we compared the cytokine profile of Th cells from pediatric malaria patients with the profile from Th cells of age- and sex-matched healthy controls (Fig. 4B). Th cells of malaria patients displayed significantly higher levels of *IFNG*, *IL10*, *IL4*, *IL17*, and *IL21*, while there was no significant increase in *IL13* expression compared with the expression in control Th cells. *TGFB* mRNA levels, in turn, were significantly lower in malaria patients’ Th cells. We then stimulated peripheral blood B cells with these cytokines as well as with CD40L, another important Th cell stimulus (Fig. 4C). In addition, we assessed the effect of hemozoin, the metabolic product of *P. falciparum* hemoglobin digestion that has been shown to upregulate AID expression [27]. While we could confirm *AICDA* upregulation by hemozoin and Th cell-derived factors known to upregulate AID, none of these stimuli lead to an increase in *APOBEC3A*.

### APOBEC3 enzymes are unlikely to contribute to mutagenesis in BL driver genes

**Figure 5:**
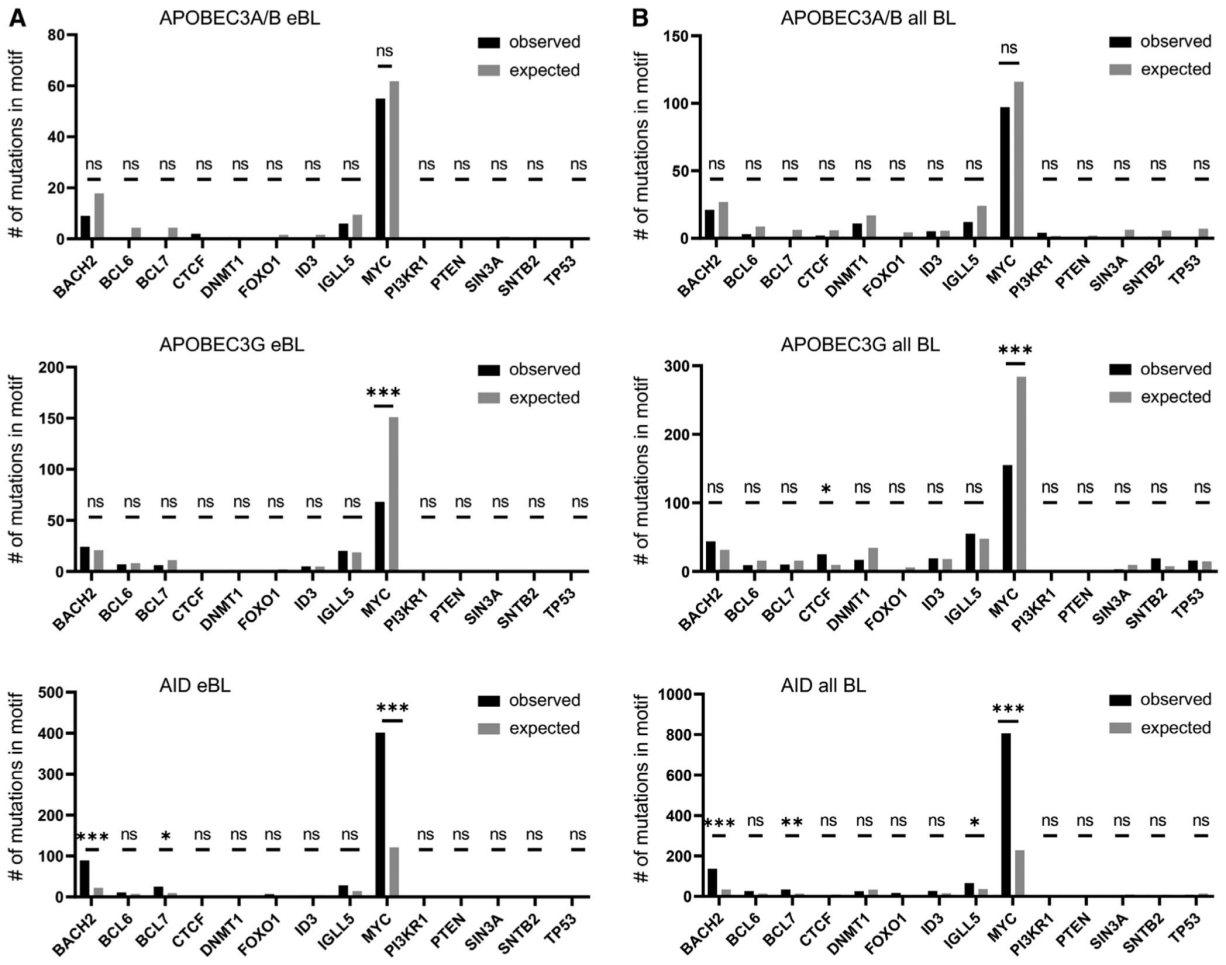
APOBEC3, APOBEC3B, and APOBEC3G do not significantly contribute to mutagenesis in BL driver genes. Coding and noncoding mutations in driver genes derived from the COSMIC database were assessed for number of mutations in APOBEC motifs, and numbers were compared to expected numbers. The following motifs and their reverse complements were analyzed: APOBEC3A/B (TCW), APOBEC3G (CCN), and AID (RCY). *p* values were calculated using Fisher’s exact test. The Benjamini-Hochberg method was used to correct for multiple comparison across all genes and motifs. Number of observed (black) and expected (grey) mutations in the APOBEC3A/B motif (top), in the APOBEC3G motif (middle), and in the AID motif (bottom). *p < 0.05, **p < 0.01, ***p < 0.001; ns, not significant. **(A)** Motif enrichment analysis considering only eBL cases. **(B)** Motif enrichment analysis considering all BL cases. Data shown are from three independent experiments.

Next, we wanted to investigate whether the AID/APOBEC enzymes induced by EBV or *P. falciparum* contribute to mutagenesis in BL. For this, we analyzed the enrichment of AID/APOBEC-derived mutations in well-described BL driver genes [36–41] by assessing the number of mutations in the APOBEC3A/B and APOBEC3G motifs and comparing them to the expected number. As a positive control, we assessed mutations in the AID motif based on the described activity of AID on various BL driver genes. In fact, when analyzing mutations considering only cases of *endemic* BL, we detected a significant enrichment of the AID motif in *BACH2*, *BCL7A*, *IGLL5*, and *MYC* mutations (Fig. 5A). The number of the observed mutations in APOBEC3A/B and APOBEC3G motifs, in contrast, was equal or even lower than the expected number. Similar results were obtained when including *sporadic* and *undefined* BL cases in the analysis, with the exception of a significant APOBEC3G motif enrichment in *CTCF*. These *CTCF* mutations, however, were predominantly derived from patients with *sporadic* BL, a BL subtype that is less stringently associated with EBV and not linked to malaria (Fig. 5B) [42]. Together, these analyses suggest that *P. falciparum*- and EBV-induced APOBEC3A, APOBEC3B, and APOBEC3G are unlikely to play a significant role in pathogenic mutagenesis in eBL.

### APOPEC3A, APOPEC3B, and APOPEC3G can target the c-MYC gene

**Figure 6:**
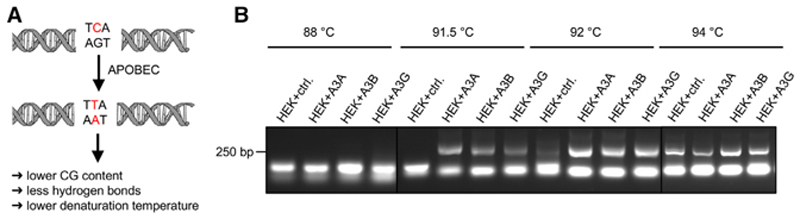
APOBEC3A, APOBEC3B, and APOBEC3G have the capacity to mutate the *c-MYC* gene. **(A)** Mutation by an AID/APOBEC enzyme lowers the amount of hydrogen bonds of the affected stretch of DNA and thereby decreases the minimal denaturation temperature required for a successful PCR reaction. (**B)** HEK293T cells were transiently transfected with APOBEC3A, APOBEC3B, APOBEC3G, or a control vector (ctrl.) and DNA was extracted after 72 h. Then, a nested PCR for the *c-MYC* gene with a gradient at the denaturation temperature was performed, the PCR product was analyzed by gel electrophoresis and the mutations were confirmed by sequencing; bp, basepair. Data shown are from three independent experiments.

Given the absence of a widespread mutagenesis by APOBEC3A, APOBEC3B, and APOBEC3G in BL driver genes despite their enzymatic activity in B cells, we wondered whether these enzymes had the capacity to recognize and mutate BL driver genes. To do so, we employed the fact that mutagenesis by APOBEC3 family enzymes leads to G/T transitions, which can be detected by differential DNA denaturation (3D)-PCR due to a reduced denaturation temperature in the mutated gene (Fig. 6A). Thus, we overexpressed APOBEC3A, APOBEC3B, and APOBEC3G in HEK293T cells, which do not express these enzymes or AID endogenously, and analyzed mutagenesis of the BL hallmark gene *c-MYC* by 3D-PCR (Fig. 6B). Indeed, in HEK293T cells transfected with APOBEC3A, APOBEC3B, and APOBEC3G, the *c-MYC* gene could be amplified at a lower denaturation temperature, and the G/T mutations were confirmed by sequencing. This suggests that APOBEC3A, APOBEC3B, and APOBEC3G have the capacity to mutate eBL driver genes, such as *c-MYC*, even though such an activity was not detected in patient-derived eBL tissue.

In summary, we found that EBV and *P. falciparum* induced the expression of various APOBEC3 enzymes similar to their previously reported induction of AID. Interestingly, however, while APOBEC3A, APOBEC3B, and APOBEC3G demonstrated enzymatic activity in vitro, they do not, in contrast to AID, seem to contribute to BL-driving mutagenesis.

## Discussion

Here, we show that EBV and *P. falciparum* malaria induced the expression of several APOBEC3 family members in human B cells. While APOBEC3A, APOBEC3B, and APOBEC3G caused mutations in the *c-MYC* gene when overexpressed in HEK293T cells, APOBEC3A-, APOBEC3B-, and APOBEC3G-derived drivergene mutations were—in contrast to AID-derived mutations—not enriched in eBL tumors. Thus, even though AID and APOBEC3A, APOBEC3B, and APOBEC3G are upregulated as part of an EBV- and malaria-directed immune response, BL-driving mutations are predominantly caused by AID, whereas upregulation of APOBEC3A, APOBEC3B, and APOBEC3G does not entail onco-genic side-effects. These findings may have substantial implications for preventive strategies aiming at targeting EBV- or malaria-related mutagenesis in eBL.

An enrichment of APOBEC3A/B and APOBEC3G motifs in BL may have been detected by using a whole genome analysis. We, however, chose to focus our analysis on previously described driver genes [36–41] to directly link mutagenesis to pathogenesis. Since the list of genes involved in BL pathogenesis is unlikely to be comprehensively defined, we cannot exclude the possibility that APOBEC3A-, APOBEC3B-, and APOBEC3G-derived mutations are relevant in yet-to-be described BL-driver genes.

Mutagenesis by AID/APOBEC enzymes is based on the presence of low-fidelity repair proteins that process the initiated DNA mismatches in an erroneous manner [5, 6]. A weak expression of the low-fidelity polymerase η together with a strong expression of the high-fidelity polymerase β has been proposed to be the reason for the relatively small number of AID-motif mutations despite considerable AID expression in precursor B-acute lymphoblastic leukemia cells [43]. Thus, the absence of widespread APOBEC3A/B and APOBEC3G mutagenesis in BL samples may be due to an insufficient presence of low-fidelity repair pathway components in human B cells that seem to be present in HEK293T cells. Similarly, APOBEC3B mutation frequency in certain cancers was reported to correlate with the expression of downstream repair pathway components, such as *REV1* and *UNG*, rather than with *APOBEC3B* levels [44], reinforcing our observation that APOBEC3 expression does not necessarily imply an APOBEC3 mutational signature.

Interestingly, the EBV protein BORF2 has been shown to relocate nuclear APOBEC3B to the endoplasmatic reticulum in U2OS, HeLa, 293T, and AGS cells, thereby preventing its mutagenic activity on DNA [11]. While it is unknown whether nuclear export of APOBEC3B takes place in EBV-infected B cells as well, such a mechanism may explain why EBV-induced APOBEC3B plays a mutagenic role in some cancers but not in others. Importantly, APOBEC3 mutagenesis is enriched in EBV-positive gastric cancer [45]. Thus, while mutagenic processes downstream of APOBEC3-mediated deamination seem to be prevented in eBL by a yet to be identified mechanism, they do take place in other EBV-related malignancies, underlining the relevance of our finding that EBV induces expression and activity of APOBEC3B and APOBEC3G.

Despite the significant increase in *APOBEC3A* expression in patients with acute malaria, the upregulation upon stimulation with EV derived from *P. falciparum*-infected erythrocytes was rather low. This suggests that even though surface proteins on malaria-infected erythrocytes seem to contribute to *APOBEC3A* induction, additional mediators are likely to play a role as well. Importantly, however, the highly significant dose-response of *APOBEC3A* expression strongly supports a role for *P. falciparum* in *APOBEC3A* induction.

Due to deferred diagnosis and limited access to treatment, malaria-related eBL is still a major health concern in sub-Saharan Africa. Our research reveals novel mechanisms of APOBEC3A, APOBEC3B, and APOBEC3G upregulation and activity, and suggests that strategies with the aim to prevent malaria-associated mutagenesis should focus on AID. Research on EBV- and malaria-induced APOBEC3 enzymes, in turn, may concentrate on a better understanding and potential exploitation of their immune-protective role.

## Materials and methods

### Healthy subjects

Cells were isolated from peripheral blood and/or palatine tonsils of healthy children undergoing routine tonsillectomy at the University Children’s Hospital Zurich.

### Malaria patients

A total of 45 children meeting the clinical criteria for acute malaria as well as having a positive malaria rapid test (Binax NOW) were enrolled at Kitgum General Hospital, Uganda, after having obtained informed parental consent. Five patients were excluded due to an insufficient amount of RNA. For patient information, see Supporting information Table S1.

### Isolation of primary cells

Primary human tonsillar mononuclear cells (TMC) were isolated from palatine tonsils obtained from pediatric patients who underwent tonsillectomy due to tonsillar hyperplasia. Briefly, tonsils were cut into small pieces with a scalpel in PBS and passed through a 70 μm-pore-size cell strainer (Falcon, Wohlen, Switzerland). Then, TMC were purified by density gradient centrifugation with Ficoll–Paque Premium (VWR International-GE Healthcare, Dietikon, Switzerland). Tonsillar B cells (TBC) were isolated from TMC using the B-cell isolation kit II according to the instructions of the manufacturer (Miltenyi Biotech, Bergisch Gladbach, Germany). B-cell and CD4+ T-cell isolation from peripheral blood of pediatric malaria patients and pediatric healthy controls was carried out using the EasySep HLA Chimerism Whole Blood CD19 Positive Selection Kit (Stem Cell Technologies) and the EasySep Human Whole Blood CD4 Positive Selection Kit (Stem Cell Technologies), as previously described [46]. Cell pellets were stored in DNA/RNA shield (Zymo Research) until RNA isolation, as previously described [46]. Peripheral B cells from adult healthy donors were isolated from Buffycoats. After 1:2 dilution with PBS, PBMCs were purified by density gradient centrifugation with Ficoll–Paque Premium (VWR International-GE Healthcare). B cells were isolated from PBMC using the B-cell isolation kit II according to the instructions of the manufacturer (Miltenyi Biotech).

### Cell culture

All cells were maintained in RPMI-1640 medium (Sigma-Aldrich, Buchs, Switzerland) supplemented with 10% heat-inactivated (hi) fetal bovine serum (FBS; Life Technologies-Thermo Fisher Scientific, Reinach, Switzerland), 2 mM L-glutamine, and 100 U/mL penicillin, and 100 μg/mL streptomycin, referred to hereafter as cRPMI. All cells were cultured at 37°C in 5% CO_2_ air with a relative humidity >95%. B cells were stimulated with recombinant human IL-4 (100 ng/mL), IL-10 (100 ng/mL), IL-13 (100 ng/mL), IL-17A(100 ng/mL), IL-22 (100 ng/mL), TGF-β1 (10 ng/mL), and IFN-γ (50 ng/mL, all Peprotech). Stimulation with HA-tagged recombinant human CD40 Ligand (CD40L; 500 ng/mL) was complemented with anti-HA antibody (200 ng/mL, both R&D Systems) enabling multimer formation. Hemozoin (LabForce AG) was used at 200 μg/mL.

### Determination of parasite load by nested qRT-PCR

DNA was eluted from dried blood spots using the QIAGEN Mini Kit (Qiagen GmbH, Hilden, Germany) according to the protocol. Punches from a clean blotting paper were run as a negative control. Parasite load was determined by nested qRT-PCR as previously described [47].

#### Preparation of virus stock and quantification

The EBV-infected marmoset B95-8 was seeded at a density of 10^6^ cells per milliliter and was stimulated to release virus by culture for 6–7 days in cRPMI containing 50 ng/mL of 12-oetradecanoylphorbol-13-acetate (Sigma-Aldrich) per milliliter. Cell suspensions were centrifuged at 1000×*g* for 10 min. Supernatant was passed through a 0.45 μm-pore-size cellulose acetate filter (Sarstedt, Sevelen, Switzerland) and stored at –80°C. Concentrated virus stocks were prepared by centrifugation of viral supernatant at 30 000 × rpm for 2 h at 4°C and resuspension of the virus pellet in complete medium (1/100 of the starting volume) and storage at –80°C.

#### Infection of primary B cells with EBV

TBC were centrifuged and resuspended in cRPMI at a concentration of 2 × 10^6^ cells/mL and EBV was added at an MOI of 8, and the cells were seeded on a 96-well plate.

#### Isolation of EV

EV from *P. falciparum* infected and noninfected erythrocytes were isolated from cell culture supernatants as described [32, 48].

### Transient transfections

HEK293T cells were transfected with AID (NM_001100779), APOBEC3A (NM_145699), APOBEC3B (NM_004900), and APOBEC3G (NM_021822) (all pCMV6-Entry vectors, OriGene Technologies, Rockville, ND) using Jet PRIME (Polyplus Transfection, New York, NY) according to the manufacturer’s protocol.

### RNA extraction, reverse transcription, and qRT-PCR

RNA was isolated using the RNeasy Mini Kit (Qiagen, Hombrechtikon, Switzerland) and was subsequently treated with DNAse (Thermo Fisher Scientific) according to the manufacturer’s protocol. For RNA isolation of cells derived from malaria patients and the corresponding controls, the Quick-RNA Micro Prep (Zymo Research) was used as previously described [46]. A total of 1 μg RNA was reverse transcribed using the High-Capacity cDNA Reverse Transcription Kit (Applied Biosystems, Foster City, CA, USA). qRT-PCR was performed using TaqMan Gene Expression Master Mix and primers and probes specific for the target genes: *AICDA* (Hs00757808_m1), *APOBEC3A* (Hs00377444_m1), *APOBEC3B* (Hs00358981_m1), *APOBEC3C* (Hs00819353_m1), *APOBEC3D/E* (Hs00537163_m1), *APOBEC3F* (Hs04184583_m1)*, APOBEC3G* (Hs01043989_m1), *APOBEC3H* (Hs00419665_m1), *IFNG* (Hs00989291_m1), *IL4* (Hs00174122_m1), *IL10* (Hs00961622_m1), *IL13* (Hs00174379_m1), *IL17A* (Hs00174383_m1), *IL21* (Hs00222327_m1), *TGFB1* (Hs00998133_m1), and for the housekeeping gene *hydroxymethylbilane synthase* (*HMBS;* Hs00609297_m1), all from Thermo Fisher Scientific. Samples were measured in technical triplicates with the 7900 Fast Real-Time PCR System (Thermo Fisher Scientific). Data were analyzed using the SDS2.2 software (Applied Biosystems) and *HMBS* was used to normalize *Ct* values.

#### Cell fractionation and deaminase activity assay

### Cell fractionation

A total of 1 × 10^7^ cells were incubated in 500 μL hypotonic solution (Hepes [10 mM, Sigma Life Science], KCl [10 mM, Sigma Life Science], MgCl_2_ [1.5 mM, Sigma Life Science], DTT [1 mM, Sigma Life Science], 1× Protease inhibitor [Roche]) for 30 min at 4°C. The cell suspension was passed six times through a 29-gauge needle and then centrifuged at 600 *g* for 1 min. The supernatant (cytoplasmic fraction) was stored. The pellet was then lysed in 500 μL GST lysis buffer (Hepes [25 mM, Sigma Life Science], glycerol [10%, Sigma Life Science], NaCl [150 mM, Sigma Life Science], Triton X-100 [0.5%, Sigma Life Science], EDTA [1 mM, Thermo Fisher Scientific], and sonicated for 3 × 5 s at 40%amplitude. After centrifugation, the supernatant (nuclear fraction) was collected.

### Western blotting

To verify the purity of the fractions, the nuclear and cytoplasmic fractions were separated on 4–12% NuPage Gels (Thermo Fisher Scientific) and transferred to nitrocellulose membranes (GE Healthcare Life Science), followed by the incubation with primary antibodies against α-tubulin (Cell Signaling Technologies, #3973, 1:2000) and Lamin A/C (Sigma Life Science, #SAB4200236, 1:1000). Primary antibodies were detected using HRP-labeled secondary antibodies (Cell Signaling Technologies) and Amersham ECL Western Blotting reagent (GE Healthcare Life Science) or Supersignal West Femto Maximum Sensitivity Substrate (Thermo Fisher Scientific) using a ChemiDoc Imaging System (Bio-Rad Laboratories; Supporting information Fig. S1).

### Fluorescence-based DNA deaminase activity assay

The nuclear fractions were incubated with 15 μL of 50 mM tris-Cl (Sigma Life Science), 10 mM EDTA (Thermo Scientific), 10 pmol of the respective oligonucleotide (Microsynth, Supporting information Table S2), and 0.02 U of UDG (Uracil-DNA Glycosylase, New England BioLabs) at 37°C for 2 h. Then, the samples were treated with 100 mM NaOH (Sigma Life Science) for 30 min at 37°C to break the DNA backbone at the abasic sites. To neutralize the reaction, 3 μL of 4 N HCl and 37 μL of 2 M tris-Cl were added. Fluorescence was measured using a Cytation imaging reader (BioTek) with excitation at 490 nm and emission at 520 nm, and the data were analyzed with the Gen5 Software (BioTek).

### Nested differential DNA denaturation (3D) PCR

Genomic DNA was isolated using the QIAamp DNA Blood Mini Kit (Qiagen GmbH, Hilden, Germany) according to the manufacturer’s protocol. First-round PCR was performed with 100 ng of genomic DNA as a template using the Taq DNA polymerase, Taq buffer, dNTP mix (all Thermo Scientific, Vilnius, Lithuania) and 1 μM forward and reverse primers (Microsynth, Table 3). The samples were amplified for 35 cycles in the SimpliAmp Thermal Cycler (Applied Biosystems by Thermo Fisher Scientific, Carlsbad, CA, USA) using an annealing temperature of 57°C. Successful first-round amplification was checked by agarose gel electrophoresis. The product from first-round PCR was purified using the QIAquick PCR Purification Kit (Qiagen GmbH, Hilden, Germany) according to protocol, and DNA was quantified using the PowerUp SYBR Green Master Mix kit with the Dual-Lock DNA Polymerase (Applied Biosystems by Thermo Fisher Scientific) and the nested (internal) primers used for the second-round PCR according to the manufacturer’s protocol. The samples were amplified in the SimpliAmp Thermal Cycler (Applied Biosystems by Thermo Fisher Scientific) using an annealing temperature of 60°C. Concentration of all samples was adjusted to the lowest concentrated samples and the second-round PCR was performed using the Taq DNA polymerase, Taq buffer, dNTP mix (all Thermo Scientific) and 30 nM forward and reverse primers (Microsynth, Supporting information Table S3). The samples were amplified for 35 cycles in the SimpliAmp Thermal Cycler (Applied Biosystems by Thermo Fisher Scientific) using an annealing temperature of 57°C and a gradient for the denaturation temperatures as indicated in Fig. 6. Finally, 5 μL of the final was analyzed by electrophoresis on 1.5% agarose gels.

### Motif enrichment analysis

Coding and noncoding mutations acquired by genome-wide screens in BL were downloaded from the COSMIC database (https://cancer.sanger.ac.uk/cosmic/download) [49]. After exclusion of SNPs, single nucleotide variants in the previously described [36–41] BL driver genes *BACH2, BCL6, BC7A, CTCF, DNMT1*, *FOXO1*, *ID3*, *IGLL5*, *MYC*, *PI3KR1*, *PTEN*, *SIN3A*, *SNTB2*, and *TP53* were analyzed for their 3 bp context. Mutation frequency within and outside the target motifs for APOBEC3A/B (TCW C = mutated base), APOBEC3G (CCN), and AID (RCY) as well as their reverse complement were compared to the frequency expected by random mutation distribution (motif frequency in the gene sequence). Fisher’s exact test and Benjamini–Hochberg correction for multiple comparison were performed to analyze the enrichment of APOBEC motif mutations.

## Supplementary Material

Supplementary figure 1

Supplementary table1

## Data Availability

The data that support the findings of this study are available from the corresponding author upon reasonable request.

## Abbreviations

AID/APOBEC: activation-induced cytidine deami-nase/apolipoprotein B mRNA editing catalytic polypeptide-like
eBL: Endemic Burkitt lymphoma
EBV: Epstein–Barr virus
EV: extracellular vesicles
FBS: fetal bovine serum
TBC: tonsillar B cells
TMC: tonsillar mononuclear cells
