# Supplementary material for: Epstein–Barr virus and malaria upregulate AID and APOBEC3 enzymes, but only AID seems to play a major mutagenic role in Burkitt lymphoma
Source: Eur J Immunol. Author manuscript; Available in PMC 2022 Aug 27. (PMC7613445; doi:10.1002/eji.202249820)
Supplement: Supplementary figure 1 [file EMS152555-supplement-Supplementary_figure_1.pdf]

Supplementary Figure S1

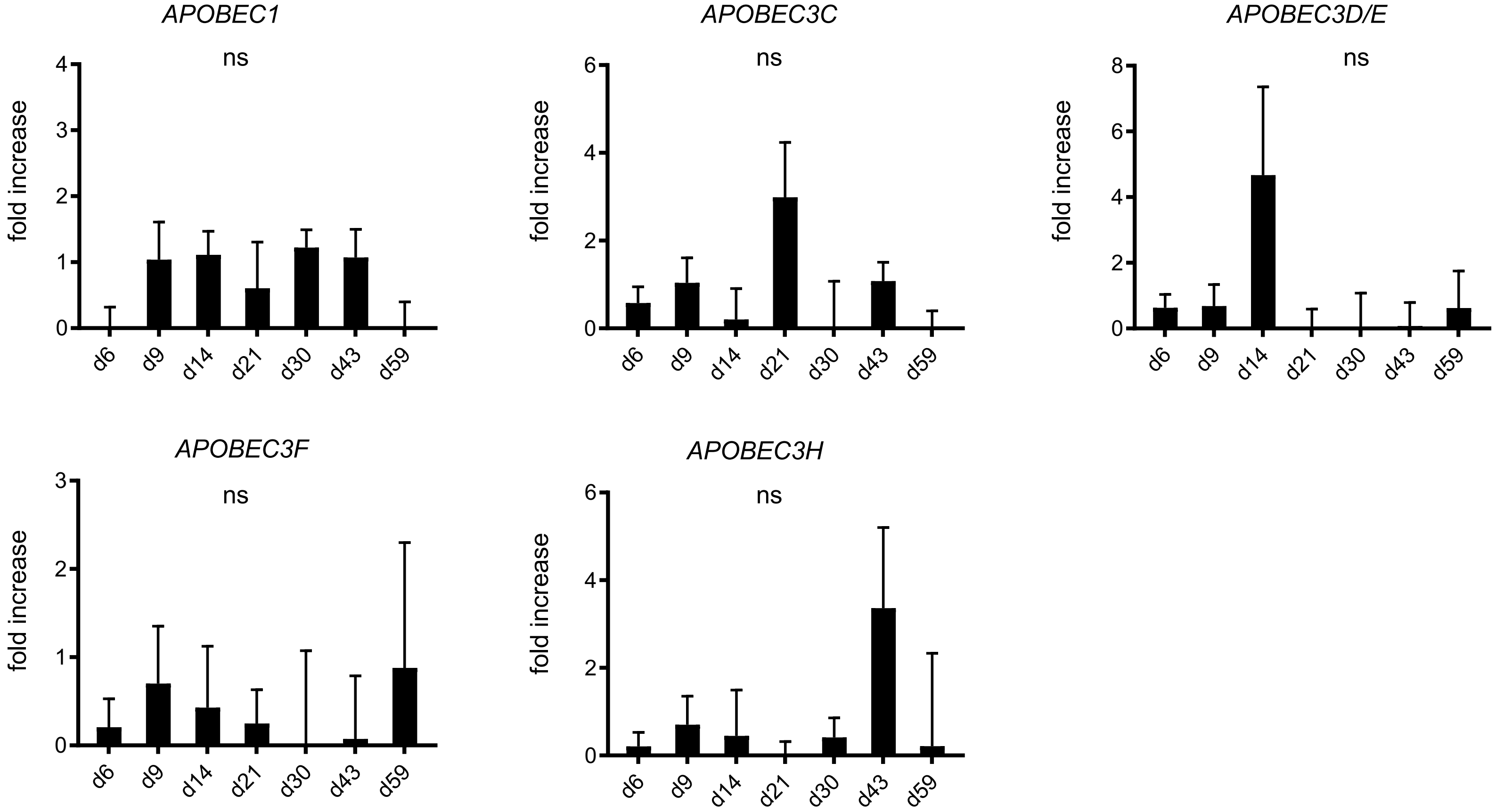

Supplemental Figure S1: EBV does not induce mRNA expression of *APOBEC1*, *APOBEC3C*, *APOBEC3D/E*, *APOBEC3F*, and *APOBEC3H* in human B cells. Tonsillar B cells were inoculated with EBV and expression of AID/APOBEC enzymes was determined by qRT-PCR. Shown is fold increase to expression in non-infected B cells of the same donor (mean+/-SD of 12 donors). P values were determined using Wilcoxon matched-pairs signed rank test with Bonferroni correction for multiple comparison; ns = not significant (there were no significant differences observed between any of the timepoints) .

Supplemental Figure S2

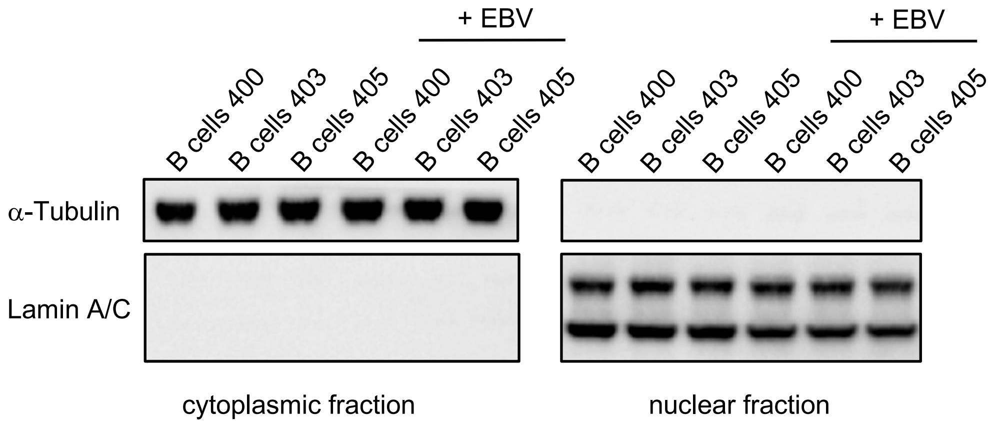

Supplemental Figure S2: Purity control of the cell fractionation for deaminase activity assays. The cytoplasmic and the nuclear fractions were analyzed for purity by Western blot using α-Tubulin (indicating cytoplasmic proteins) and Lamin A/C (indicating nuclear proteins) antibodies.

Supplementary Figure S3

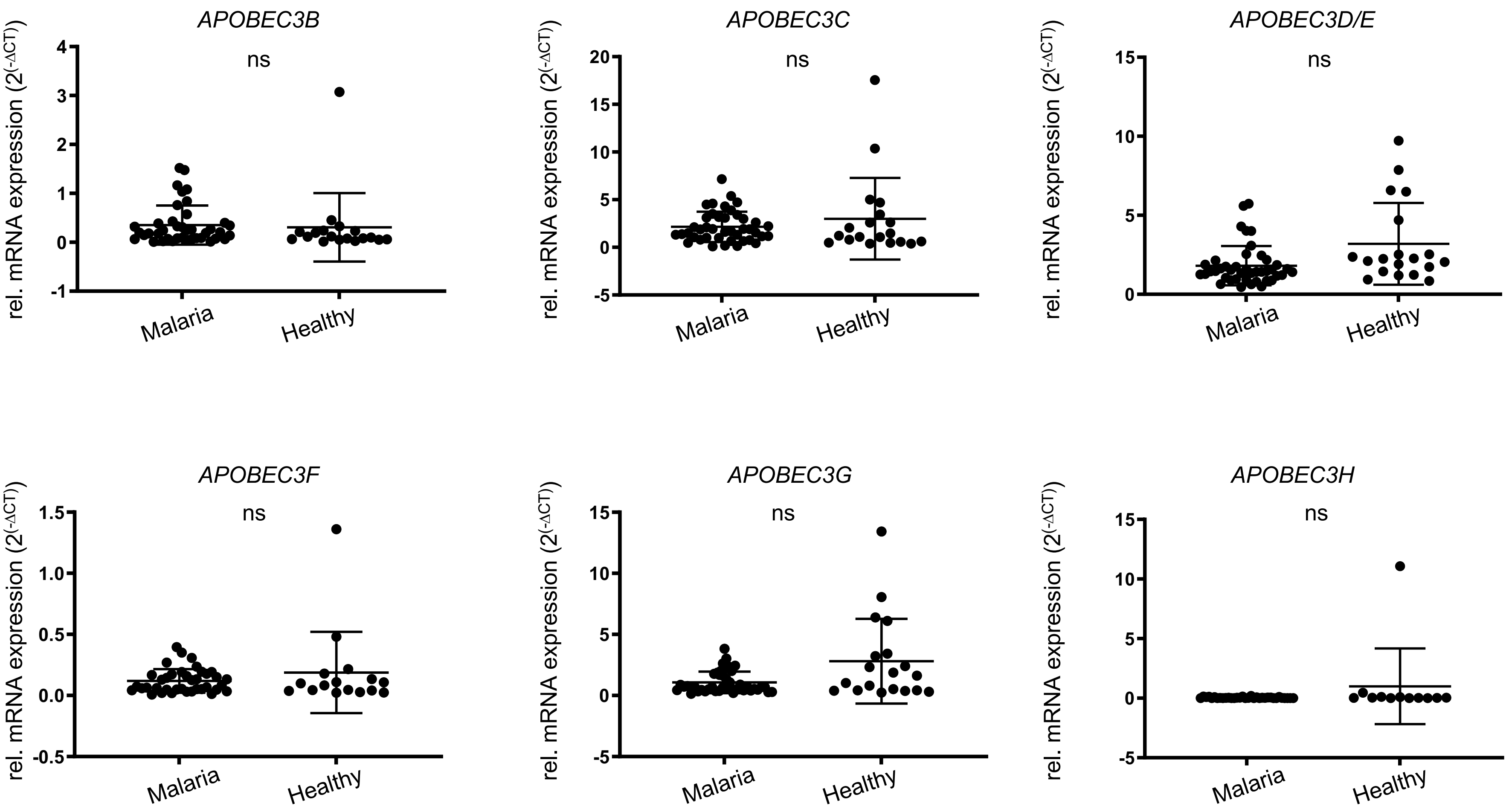

Supplemental Figure S3: *P. falciparum* does not induce mRNA expression of *APOBE3B*, *APOBEC3C*, *APOBEC3D/E*, *APOBEC3F*, *APOBEC3G*, and *APOBEC3H* in human B cells. Expression of AID/APOBEC enzymes in B cells of malaria patients and age-matched healthy controls was measured by qRT-PCR. Dots represent individual donors (N=40), bars represent mean +/- SEM, $\Delta CT = CT^{AID/APOBEC} - CT^{HBMS}$. P values were determined using Mann-Whitney test; ns = not significant.
