# Supplementary material for: Epstein–Barr virus and malaria upregulate AID and APOBEC3 enzymes, but only AID seems to play a major mutagenic role in Burkitt lymphoma
Source: Eur J Immunol. Author manuscript; Available in PMC 2022 Aug 27. (PMC7613445; doi:10.1002/eji.202249820)
Supplement: Supplementary table1 [file EMS152555-supplement-Supplementary_table1.docx]

**SUPPLEMENTAL TABLES**

**Supplemental Table 1**

| Age (years) | Range: 0.42 – 10  Mean: 4.3  SEM: 2.6 |
| --- | --- |
| Sex | 24 m, 21 f |
| Temperature (°C) | Range: 35.6 – 40.6  Mean: 37.10  SEM: 2.6  Number of patients >37.5: 15 |
| Malaria severity according to WHO World malaria report 2017 | Severe: 4, non-severe 41 |
| HIV status | Positive: 0 negative: 45 |
| EBV status | Positive: 39 negative: 6 |
| Malaria medication at time of blood sampling | Yes: 14 No: 31 |

**Supplemental Table 1:** Patient information.

**Supplemental Table 2**

| **Specificity** | **5’ Modif.** | **5’-3’ Sequence** | **3’ Modif.** |
| --- | --- | --- | --- |
| **A3A/B** | FAM | AAATTCTAATAGATAATGTGA | TAMRA |
| **A3G** | FAM | AAACCCAAATAGATAATGATAATGTGA | TAMRA |

**Supplemental Table 2:** Oligonucleotides used in fluorescence-based DNA deaminase activity assay

**Supplemental Table 3**

|  | **5’-3’ Sequence** |
| --- | --- |
| **First-round forward** | ATG CCC CTC AAC GTT AGC TTC ACC AA |
| **First-round reverse** | CTG GAT GAT GAT GTT TTT GAT GAA |
| **Second-round forward** | ACG AGG AGG AGA ACT TCT ACC AGC A |
| **Second-round reverse** | TCT CGT CGT CCG GGT CGC AGA TGA A |

**Supplemental Table 3:** Primers used for nested 3D PCR
